# An ethnographic study of strategies to support discussions with family members on end-of-life care for people with advanced dementia in nursing homes

**DOI:** 10.1186/s12904-016-0127-2

**Published:** 2016-07-07

**Authors:** Geena Saini, Elizabeth L. Sampson, Sarah Davis, Nuriye Kupeli, Jane Harrington, Gerard Leavey, Irwin Nazareth, Louise Jones, Kirsten J. Moore

**Affiliations:** Centre for Mental Health, Maya House, 134-138 Borough High Street, London, SE1 1LB UK; Marie Curie Palliative Care Research Department (MCPCRD), Division of Psychiatry, University College London, 6th Floor, Maple House, 149 Tottenham Court Road, London, W1T 7NF UK; Bamford Centre for Mental Health & Wellbeing, University of Ulster, Magee Campus, Northland Road, Derry Londonderry, BT48 7JL UK; Department of Primary Care and Population Health, UCL Royal Free Site, Rowland Hill Street, London, NW3 UK

**Keywords:** Advanced dementia, End-of-life discussions, Nursing homes, Family carers, Ethnography

## Abstract

**Background:**

Most people with advanced dementia die in nursing homes where families may have to make decisions as death approaches. Discussions about end-of-life care between families and nursing home staff are uncommon, despite a range of potential benefits. In this study we aimed to examine practices relating to end-of-life discussions with family members of people with advanced dementia residing in nursing homes and to explore strategies for improving practice.

**Methods:**

An ethnographic study in two nursing homes where the *Compassion Intervention* was delivered. The *Compassion Intervention* provides a model of end-of-life care engaging an Interdisciplinary Care Leader to promote integrated care, educate staff, support holistic assessments and discuss end of life with families. We used a framework approach, undertaking a thematic analysis of fieldwork notes and observations recorded in a reflective diary kept by the Interdisciplinary Care Leader, and data from in-depth interviews with 23 informants: family members, GPs, nursing home staff, and external healthcare professionals.

**Results:**

Four major themes described strategies for improving practice: (i) educating families and staff about dementia progression and end–of-life care; (ii) appreciating the greater value of in-depth end-of-life discussions compared with simple documentation of care preferences; (iii) providing time and space for sensitive discussions; and (iv) having an independent healthcare professional or team with responsibility for end-of-life discussions.

**Conclusions:**

The Interdisciplinary Care Leader role offers a promising method for supporting and improving end-of-life care discussions between families of people with advanced dementia and nursing home staff. These strategies warrant further evaluation in nursing home settings.

## Background

Approximately 850,000 people in the UK and 47.5 million worldwide have dementia [1, 2]. The clinical course of advanced dementia is unpredictable [3] and planning for end-of-life (EOL) care may be difficult [4]. Despite UK policy recommending that people should receive adequate EOL care regardless of their health condition [5], people with dementia have limited access to palliative services and receive care that is often suboptimal [6–8]. Many people with advanced dementia die in nursing homes (NHs), and discussions to enable planning for care as death approaches may be useful [9]. In the UK the general consensus is that EOL planning and discussion of future wishes should begin in the early stages of dementia, before the individual loses capacity [10, 11]. However, a person with dementia, even in early stages, may have difficulties considering their preferences for future care [12].

### Benefits of EOL care discussions

A systematic review of research conducted mostly in the USA in hospital and nursing home settings showed that when EOL discussions occurred, the person dying with dementia experienced fewer aggressive and unnecessary medical interventions [13]. Those with a documented Advance Care Plan (ACP) had fewer calls to emergency services, hospital admissions, bed days and costs [14, 15]. Discussing EOL care may result in fewer burdensome interventions in nursing homes for residents with cognitive impairment [15, 16].

EOL discussions may have benefits for families. A trial in the USA found that EOL conversations and a follow-up telephone conversation with family members of NH residents with dementia led to increased family satisfaction with care and better documentation of preferences for future care [17]. Conversations of at least fifteen minutes duration with family members on admission of their relative with dementia into a NH may improve satisfaction with EOL care [18] and provide families with the opportunity to clearly think about which treatment may or may not be helpful [14, 17, 19, 20].

NH staff may benefit from improved communication with families. For example, knowledge of family and individual preferences may improve staff confidence in formulating care decisions as death approaches [21]. However, ACP and documented discussions of preferences for future care often do not occur in practice. Results of a UK survey of 213 NH managers made available in 2009 found that while 89 % required or recommended an ACP process in their NH less than 25 % of residents had a documented ACP in place [22].

### Barriers to care discussions

Commonly, families do not perceive dementia as a life limiting condition [23, 24] and with-holding treatment can be morally and emotionally difficult [23, 25, 26]. Discussions on EOL care may highlight anticipatory grief for families who may feel they lack support [27]. Family carers may be ambivalent regarding what is an appropriate level of EOL treatment and many do not want to think about their relative’s death [28, 29]. Family carers of people with advanced dementia in nursing homes describe feeling ill-prepared and unsupported in making EOL decisions for their relatives [30]. Communication between the family member, the individual with dementia and a respected and knowledgeable professional may help families during this difficult process [28]. NH staff, however, often lack confidence in initiating EOL discussions [31] and report a need for greater understanding of the nature and likely outcomes of dementia, while acknowledging their own limitations in discussing EOL care [21]. They may be reluctant to discuss the possibility of approaching death due to fears they may be considered accountable in some way if the person dies [32]. There may be some resistance to discussions from families who are struggling to accept that their relative may die soon [32, 33]. Even when families have expressed EOL decisions, these may not be followed if physicians feel for example that resuscitation is inappropriate [26] or NH staff feel vulnerable about being blamed for “poor care” and wish to transfer a resident to hospital [32]. One UK study of NH staff showed that some believe it is their role to “preserve life as long as possible” sending the individual to hospital even if this is contrary to the wishes of family members [32].

Many healthcare professionals (HCPs) tend to view EOL discussions as “someone else’s problem” [34]. There is debate regarding who is most appropriate to lead discussions, some suggest General Practitioners (GPs), palliative care specialists, dementia nurse specialists (such as Admiral Nurses in the UK) or a new role [35, 36]. These uncertainties and the doubts about appropriate timing for discussions can delay them [36].

### Rationale and aim

In this study, we aimed to examine practices relating to end-of-life discussions with family members of people with advanced dementia residing in nursing homes and to explore strategies for improving practice. We report observations noted by an Interdisciplinary Care Leader (ICL) embedded in two NHs to implement the Compassion Intervention, the *‘Intervention’* [37]. We combined observations recorded by the ICL with qualitative interview data from HCPs and families on their experiences of care.

## Methods

We conducted an ethnographic study using qualitative data extracted from a reflective diary kept by a professional delivering the *Intervention* (ICL) and from transcripts of interviews with HCPs and family members of people with advanced dementia residing in NHs.

### The *Compassion Intervention*

Our research team developed the *Intervention* as part of a 3 year research programme on EOL care in advanced dementia [38]. The *Intervention* is delivered by an ICL and has two core components: (i) facilitation of integrated care for people with advanced dementia and (ii) training and support for those working with and caring for people with advanced dementia. This has been implemented in an exploratory naturalistic study [39].

### Interdisciplinary care leader

An ICL was employed full time to work in two NHs located in two Clinical Commissioning Groups (responsible for health and social care funding allocation in local areas) in Greater London for six months. The ICL had a social science background with considerable experience of working with people with severe dementia in NHs. She was trained and received supervision by clinicians with expertise in palliative care, dementia care and primary care. The ICL was present in each NH for three half-days per week and undertook the tasks described below.

### Intervention component 1: facilitation of integrated care

The facilitation of integrated care aimed to promote engagement with healthcare professionals from a mix of disciplines required to meet the diverse needs of residents with advanced dementia. Previous studies have identified that NH residents face barriers in accessing healthcare services [40]. Residents eligible for receipt of the *Intervention* were those with advanced dementia [39] identified by NH staff. The ICL completed holistic assessments of residents which included discussions with family members and staff, review of the care plan and discussions with or observations of residents. The ICL attended weekly meetings with NH nurses and when possible the GP supporting the NH. In these meetings residents’ care needs were discussed, the need for external referral reviewed and EOL plans agreed. Wider multidisciplinary team (MDT) meetings involved the ICL with NH nurses, managers, external geriatricians, GPs, mental health and palliative care and were conducted in NH1 on a monthly basis to discuss complex issues relating to palliative care and to undertake after death analysis. The ICL discussions with family covered concerns raised by the family, common symptoms in advanced dementia, EOL care and whether the family member was coping or needed more support.

### Intervention component 2: training and support for families and carers

The ICL ran formal training sessions for staff and family and informal on-the-job advice and support. Staff training sessions covered behavioural symptoms, pain management and EOL, and family sessions covered the trajectory of dementia, common EOL symptoms and the personal experiences of care. Further details of the *Intervention* are published [39].

### Nursing home settings

The *Intervention* was delivered in two nursing homes (NH). Nursing homes provide 24 h nursing care as well as accommodation for people who are unable to live independently. Quality standards are regulated in the UK by the Care Quality Commission. Nursing staff oversee medical care (managed by GP) including medications and liaise with residents, family, the GP, NH managers and healthcare assistants. Most healthcare assistants have minimal training either prior to or during NH employment. They support residents in care tasks including bathing, dressing, eating and social activities while activity co-ordinators also promote and organise activities and opportunities for resident social engagement. NH1 had 99 beds consisting of five units: one residential care; one rehabilitation; two nursing care and one dementia specific. Each was managed by a nurse with up to five healthcare assistants. Across all units there were approximately two full-time equivalent activity co-ordinators. The GP visited for two sessions (approximately 3 hours per session) per week. NH2 had 77 beds with three units: residential care and two nursing care units, one was dementia specific. The nursing care units were run by a nurse with up to four healthcare assistants. There was one full-time equivalent activity coordinator. The GP visited one session a week.

### Data collection

#### ICL reflective diary

The ICL’s role was ethnographic in that she was observing individuals within their natural setting, ie two NHs, to develop an appreciation of existing practices, cultures and behaviours of those living, working and interacting in this setting [41]. However, while the ICL took a stance of being respectful and appreciating the existing setting [42], she was also tasked with identifying areas where EOL care could be improved based on her observations, hence trying to influence change on the natural setting. Based on the best available evidence, the *Intervention* adopts the stance that communicating sensitively and openly with family about EOL symptoms and care is likely to support comfort care and a palliative approach at EOL for both residents and family. Through implementing the *Intervention* the ICL was raising awareness regarding potential benefits of these conversations as well as supporting staff in having discussions. The ICL therefore had a dual role involving passive observation and active intervention.

To record her observations of these two processes, the ICL recorded a reflective diary each day she visited either NH. She recorded experiences, ideas and personal reactions to delivering the *Intervention* within the NHs [43, 44]. Self-observations, self-doubts and thoughts were recorded along with how effective the ICL believed her role to be [45]. General observations and reflections on conversations with residents, staff or family were included. As the ICL role was clinical she worked according to each NH’s governance policy and was not permitted to collect identifiable research data.

### Interview data

To gain insights into the *Intervention* from the perspective of families and staff involved with the *Intervention*, we undertook semi-structured interviews using open-ended questions after 6 months of implementing the *Intervention*. As the interviews were to obtain experiences of interacting with the ICL, we felt that it would enable participants to be more open in their responses if they were not being interviewed directly by the ICL. These interviews were therefore undertaken by experienced researchers with backgrounds in medicine, nursing and psychology (NK, SD, ME). Interviews were audio-recorded, transcribed verbatim and transcripts checked before analysis. All participants who took part in an interview provided written informed consent.

#### Family carers

Eligible carers included those who were family members/key contacts of residents who had been assessed by the ICL. NH managers sent expressions of interest letters to eligible carers asking them to indicate whether they were happy to be contacted by the research team. Non-responders received two mailed reminders. If they returned the expression of interest to the research team they were then sent an information and consent form asking whether they assented for the resident to have observational monthly assessments and secondly, whether they agreed to monthly interviews to measure carer burden, grief and depression [39]. These findings will be published separately. If they did not return the consent form, the research team was allowed to ring them once as a reminder. Carers who consented for the monthly data collection were also invited to complete the interview after six months of implementing the *Intervention*. Interviews explored their experience of talking to the ICL and having EOL discussions and caring for someone with dementia as they were deteriorating.

The ICL assessed 28 residents. Seven residents did not have a key contact whom we could contact (three residents had no key contact, two residents had key contacts with incorrect contact details, and two contacts were unavailable for interview due to living in another country). Another four residents died or moved nursing home before assent, one key contact indicated that her late father requested her mother was never to participate in research, one family member indicated they had no involvement with the resident and eight key contacts did not respond to the manager’s invitation. The remaining seven key contacts (all family members; one from NH1 and six from NH2) were invited to take part in the interview. Of these seven, four (all from NH2) were interviewed at the NH, including two daughters, a husband and a son, between the ages of 54 and 76. All were white British, the employment status of two was unknown whilst one was retired and one employed. Interviews lasted approximately 10 to 25 min.

#### Staff/formal carers

Researchers approached staff at the NHs or emailed those who were based externally. The same structure and location as the family interviews was used, although some external HCPs requested the interview be undertaken in their usual workplace. Topics included: communication of the dementia trajectory to relatives, the influence of the ICL in the NH and the usefulness of the training sessions delivered by the ICL.

**Fig. 1:**
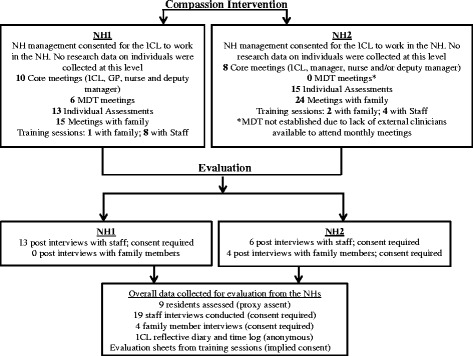
Summary of consent and sample size

Nineteen HCPs participated in interviews, 13 at NH1 and six at NH2, representing approximately 10 % of NH care staff. Various NH staff were interviewed: managers (*n* = 2), activity co-ordinators (*n* = 2), deputy managers (*n* = 3) nurses (*n* = 2) and healthcare assistants (*n* = 6). A GP from each NH and a palliative care nurse and a geriatrician who visited NH1 were interviewed. Interviews lasted between five to 35 min. Figure 1 shows the core components of the *Intervention* including the involvement of participants and consent processes.

### Data analysis

Interview transcripts were checked against the recordings to ensure accuracy [46]. The transcripts and text of the reflective diary were read thoroughly numerous times to ensure the researcher became familiar with the content and brief summary notes were made [47]. Data were analysed manually and coded using thematic analysis. Codes, which label sections of information as “meaningful units”, were manually attributed to paragraphs or lines of the data, with all data given the same amount of attention [46–48]. To ensure validity and rigour the coding process was thorough and key themes were not formed from a small number of examples within the data [46]. Identification of key themes focused on identifying areas of good practice or strategies that may help to promote EOL care discussions. After establishing the key themes, codes under each key theme were reviewed and grouped into smaller subthemes. We then tabulated these subthemes according to whether codes were obtained from the interviews, the reflective diary or both. This enabled triangulation of the data from the perspectives of the ICL, family carers and healthcare professionals and allowed us to build a picture of the themes within the context of care. GS conducted the analysis which was independently checked by two other researchers; SD conducted an independent analysis and identified codes and themes in the reflective diary and KM did the same for the interviews. KM also checked themes identified from the diary to ensure they were consistent with her experiences as ICL. ELS (old age psychiatrist) reviewed themes throughout the analysis period to check that they were clinically relevant.

## Results

We identified four key themes arising from the reflective diary and qualitative interviews. These were (i) educating family and staff about dementia progression and EOL care; (ii) appreciating the value of in-depth EOL discussions over documentation; (iii) providing time and space for sensitive discussions; and (iv) having an independent HCP or team with responsibility for EOL discussions. Table 1 shows each theme with associated subthemes and indicates whether the subtheme was identified by the ICL or through interviews.

**Table 1:** Summary table of findings by theme and whether supported in diary or interviews

Themes	Findings	Supported in diary	Supported in interviews
Educating family and staff about dementia progression and EOL care	Families and staff needing and wanting more information about diagnosis, symptoms and progression of dementia	✓	✓
	NH staff lacking confidence to initiate and have EOL conversations	✓	✓
	Staff attributing symptoms and behaviours to dementia without trying to identify an underlying cause	✓	
	Training and case scenarios increasing staff confidence and being able to see things from the families’ perspective	✓	✓
	Discussions with family appear to increase their capacity to make informed decisions, eg around cardiopulmonary resuscitation	✓	✓
	Family sessions generated much discussion and appeared a good avenue for education	✓	✓
	Usefulness of written information to support discussions		✓
	Importance of ICL as a role model to staff in having conversations with family and communicating with residents with advanced dementia		✓
Appreciating the value of in-depth EOL discussions (over documentation)	Importance of ongoing dialogue with family to build relationships, provide reassurance and allow time for family to process information	✓	✓
	NH staff prioritising documentation such as DNAR or not for hospitalisation over ongoing dialogue – task oriented approach and not appreciating the complexity and need for individualised approach to these discussions	✓	
	Importance of addressing family member’s current issues and concerns before discussing future plans	✓	
	Need to acknowledge family members’ grief and guilt	✓	
	Difficulties communicating in English prohibit in-depth and sensitive conversations about EOL	✓	✓
	Importance of information provided in a sensitive way	✓	✓
Providing time and space for sensitive discussions	Not suitable having sensitive conversations with family in communal areas such as lounge or dining room	✓	
	Spending sufficient time with family to address their questions and explore their concerns – including follow-up sessions/ongoing dialogue. The ICL was able to provide this time.	✓	✓
	NH staff and GP having multiple demands preventing spending focused and uninterrupted time with family	✓	✓
Having an independent healthcare professional or team with responsibility for EOL discussions	ICL role was independent from GP and NH and considered to be primarily in interests of resident and family	✓	✓
	Independent person provides alternative and fresh view of the residents’ needs and care		✓

### Educating family and staff about dementia progression and EOL care

Through interviews and the ICL diary it was evident that educating family and staff about the progression of dementia was essential for underpinning EOL conversations and guiding care. Eight subthemes were identified (See Table 1). Only one was identified by the ICL but not in the interviews, indicating that symptoms and behaviours associated with dementia were generally attributed to dementia in care plans. As dementia is not “treatable”, attributing symptoms to dementia removes the need to explore possible underlying causes of symptoms and behaviours that may be treatable or where the resident’s comfort and quality of life could be improved.

*…I don’t want to see things in the care plan such as the resident fell because of dementia, the resident is not eating because of dementia. I want [staff] to think a bit more deeply about what is happening to the resident and what the resident might be trying to communicate, to think of unmet needs etc. (ICL)*

Two subthemes related to staff confidence in initiating EOL conversations and included staff lacking confidence and that training and case scenarios were useful tools for increasing staff confidence and seeing things from the families’ perspective:

*One nurse said that she felt…more confident talking to family…after the case scenarios…another nurse said that she could think more from the perspective of the family member… (ICL)*

*I did gain some experience from… like I was the patient [family] and you were the nurse so I was you know… [Interviewer: role-playing]… We had role-play and I found that very, very, very, very useful. (NH2 Nurse and Deputy Manager)*

A similar subtheme which was only reported in interviews was the importance of the ICL in role modelling EOL conversations with family with NH staff present to observe:

*It's quite good…you can see how she [ICL] explains to them [family] and…the difference is between us talking to them and a professional like her… (NH1 nurse)*

Although the ICL provided individualised discussions with family members, managers at both NHs requested group sessions for family members. These sessions were positively received by family (evaluation forms not reported) and generated many questions about dementia and its progression, highlighting the need for greater dementia education to family carers.

*There was a lot of discussion… about dementia…diagnosis process…acceptance of dementia amongst family and…society…how this hindered the diagnosis process… early part about dementia identification, diagnosis, symptoms…family inheritance (ICL)*

Another related subtheme that was only raised in family interviews was the value of providing written information to support discussions:

*She [ICL] was the one who spoke to me and gave me a very good leaflet to read, the stages she would go through and that did make… it a lot clearer… So in that sense that was excellent and …she was very caring and she was the one that explained it all to me (NH2 Family member)*

A subtheme identified in the diary and interviews was that discussions with family appeared to increase their capacity to make informed EOL care decisions.

*I started telling her why this (cardiopulmonary resuscitation) can be inappropriate for someone in the advanced stages of dementia…the likelihood of it being successful was very low. She said that when you put it that way it made more sense… (ICL)*

### Appreciating the value of in-depth EOL discussions (over documentation)

The ICL diary and interviews with staff highlighted their perceptions that family members valued EOL discussions more than formalising discussions in writing. Staff, however, placed greater emphasis on documenting future wishes, possibly reflecting NH requirements to demonstrate that EOL discussions have been held. The ICL had ongoing conversations with families, communicating and providing support, as and when it was needed, and explained the importance of these discussions to staff. Six subthemes were identified (See Table 1). The one subtheme found in both the ICL diary and interviews was the importance of ongoing dialogue with family to build a trusting relationship, provide reassurance and allow time for family to process information. The needs, wishes, anxieties of family members are part of an evolving process which the ICL facilitates, whereas documented EOL plans can be seen as static and immutable and perhaps a potentially frightening commitment by many families.

*When I have plenty of time and sometimes talk to family members for well over an hour, we don’t usually get to a point where they are ready to complete an ACP or change goals of care…requires ongoing discussions…reflections…perhaps some involvement from the GP. (ICL)*

*I think just their reassurance…there is nothing physically they can do…they just reassure you…. That you are doing the right thing, more than anything, because sometimes you do doubt yourself (NH2 Family member)*

*Residents and next of kin, loved ones, they don't ever have the opportunity to talk about what to expect towards the end of um you know; the spectrum of dementia, it always comes as a surprise to them… I think with [ICL’s] involvement there was an opportunity for them to have someone to talk about that to them…I think she also gave them supplementary information in a written form for them to then go away … to digest… and then giving them a further opportunity to come back with any other concerns. (NH1 Geriatrician)*

Three subthemes were only identified in the ICL’s diary. The first was that NH staff prioritised documentation such as Do Not Attempt Resuscitation (DNAR/DNR) or “not for hospitalisation” over ongoing dialogue. This was a task-oriented approach and they often did not appreciate the complexity and need for individualised and ongoing discussions.

*I said…I had spoken to the son… that they wanted care to be provided in the care home and DNR. She said that was no good unless they had it signed. I thought that this was some progress…you can’t rush or push people to complete these… confronting plans… (ICL)*

*One nurse said that they had discussed end of life with a couple of family members but that they had refused to sign any ACP or DNR. She seemed to imply that because there was nothing documented that it hadn’t really been worth having the discussion. (ICL)*

The second subtheme only identified by the ICL was the importance of addressing family members’ current issues and concerns before discussing future plans.

*in the first scenario… the nurse was trying to talk about end-of-life care and DNRs while the ‘family member’ was talking about (as per the scenario) her concerns about the care at the care home…the nurse did not pick up and try to alleviate the family member’s concerns about the quality of care… We talked about how if she had talked more about comfort care …what was happening to the resident today and that that would have addressed the concerns that the family member was raising.(ICL)*

The final sub theme addressed only in the ICL diary was the need to acknowledge family members’ guilt and grief.

*She cried at one stage… She felt that dementia was a horrible disease and hated what it did to her loving gentle husband who was now aggressive and agitated (ICL).*

The final two themes arose from the interviews and ICL diary and related to issues regarding language and communication barriers and the importance of providing information in a sensitive way.

*I find that the nurses tend to feel they don't really know how to start the conversation. It is often a very difficult conversation for them to initiate and then even if they can initiate it is then the depth of that discussion is often lacking (NH1 Geriatrician)*

*Staff need help talking to relatives…language is a problem…none of them are English born… … haven’t got… subtleties of language, when a conversation is… difficult… it can come over a bit more blunt when … 'do you want your relatives to go to hospital… to be resuscitated or not?' They don't know how to develop those conversations. (NH1 Palliative care nurse)*

### Providing time and space for sensitive discussions

Through both the ICL diary and interviews it was seen that providing time and space to have EOL discussion was important. One subtheme was only identified in the ICL diary and indicated that it was not suitable having sensitive conversations with family in communal areas such as lounge or dining room (see Table 1).

*It is very difficult having a conversation in the main lounge with all the other residents… family members and staff in the room. (ICL)*

The remaining two subthemes were found in both the ICL diary and the interviews. The subthemes were interrelated reflecting the need for adequate time for sensitive conversations, which was enabled by the ICL, and that NH staff and GPs had difficulty managing high workloads and multiple demands.

*I think takes time; because it's not one that you can do in one sitting. That often you need to build the relationship and then go it step by step. And I think that’s where [ICL] role is quite unique in that she can come back and have a second conversation, a third conversation and a fourth if that is required (NH1 Geriatrician)*

### An independent healthcare professional or team with responsibility for EOL discussions

The last theme identified from the ICL diary and the interviews was the importance of having an independent healthcare professional to have EOL discussions with family. One subtheme from the ICL diary and the interviews reflected the value of the independence of the ICL role, one that is primarily for the interests of residents and family, rather than those of health professionals. This allowed family members more ease in talking to the ICL.

*… helpful to have someone independent of the care home – an independent voice where… family can feel more open about the care …not feel that motives of the care home are influencing the discussion. (ICL)*

*They [family] feel comfortable discussing… in an environment where they don't feel they have to actually make a decision, …I think in hospitals…when they speak to doctors, they… feel that we are trying to make them say things they might not necessarily want to say…we talk about the best interest of the patient…she is the neutral person they tend to feel a bit more comfortable having her there. (GP)*

The second subtheme, only identified in the interviews, was that an independent person provided an alternative and fresh view of the resident’s needs and care.

*We feel it’s helpful because she has got a different way of looking at the situation. The areas where we don’t normally see… it will help and improve in the care of these service users (NH1 Nurse and Deputy Manager)*

## Discussion

In this study we aimed to identify strategies for promoting EOL discussions with family members of people with advanced dementia living in NHs, using data arising from the new and exploratory role of the ICL, in combination with qualitative interviews with families and professionals. We identified four key strategies including; (i) educating family and staff about the progression of dementia and EOL care; (ii) appreciating the value of in-depth EOL discussions over simple documentation; (iii) providing time and space for sensitive discussions; and (iv) promoting an independent healthcare professional or team with responsibility for EOL discussions. Consistent with previous studies [14, 17, 20], we found EOL discussions provided an opportunity for families to learn about dementia progression, but we also found that staff needed more dementia education to enable discussions with families.

### Consistent findings between the reflective diary and interviews

Both the diary and interviews with healthcare professionals showed that family members lacked knowledge about dementia progression [49] which could be problematic if they are consulted about their relative’s EOL care but are unaware of the life limiting nature of dementia. Increased education, support and understanding of advanced dementia may offer more realistic expectations about dementia outcomes and may help increase family satisfaction with EOL care [18]. Both data sources also highlighted the use of training to increase staff confidence in discussing EOL. In a previous study [31] staff claimed that training and supervision were key to increasing their confidence when initiating ACP discussions.

### Inconsistent findings between the reflective diary and interviews

Although most themes were evident in both the diary and interview data, there were important differences. Our use of the diary data enabled us to identify cultural issues within the NHs that were not identified in the interviews. Crucially, the dissonance between families’ desires for ongoing discussions about current and future care issues, and the needs of NH staff, which tended to be task-driven and emphasised the documentation of preferences for resuscitation and hospitalisation. NH staff appeared to underestimate the importance of discussing EOL care with family members through ongoing dialogue in which trusting relationships are fostered [50].

Findings from the healthcare professional interviews that were not evident in the diary included the importance of the ICL in providing an alternative view of residents’ needs and the value of the ICL in role modelling EOL conversations for staff development. The need for supplementing verbal discussions with supporting written information was only raised by family carers, although the ICL did routinely provide written information after discussions with family members, acknowledging this as important.

### Strengths and limitations

This study’s method aimed to provide a range of approaches to improve the validity and quality of the findings. This included: adapting the *Intervention* to a real life situation; having the researcher (ICL) becoming familiar with the study context by observation and working within it; incorporating a range of views including the ICL’s, family carers’ and staff within and external to the NHs; and having multiple researchers involved in analysis [51]. Previous research has focussed on NHs [14–17, 25, 32] however this is the first study to implement the role of an ICL in a NH setting. We acknowledge the post of the ICL was unusual and that funding for such a role within usual settings may be challenging.

The ICL’s reflective diary offers a holistic, authentic picture; the ICL was the ethnographer observing the natural setting while also using these observations to inform the need for improvements in EOL care conversations. The ICL was immersed in the NHs and developed relationships with NH staff, family and residents. Participant observation enabled the ICL to see family members and staff act in their usual setting. The ICL was open to new insights as she did not have a fixed set of questions that she had to address and she was able to explore things that may not have been addressed in direct interviews or focus groups.

This study also used qualitative interviews with staff and families to allow for triangulation of data. The benefits of triangulation include “increasing confidence in research data, creating innovative ways of understanding a phenomenon, revealing unique findings, challenging or integrating theories, and providing a clearer understanding of the problem” [52]. The interviews provided verification of many of the findings in the ICL’s diary. A number of differences between the two data sources highlighted some differing views obtained from diverse perspectives. However, the volume of data available in the ICL diary outweighed the data from the staff and family interviews and therefore the findings more heavily rely on data from the diary. It should be borne in mind, however, that all the data arose from the same context of care. While our data provides perspectives from a wide range of disciplines within and external to the NH settings, the small number of participants in each discipline prevents comparisons between different disciplines. The volume of data from staff interviews also outweighed data from family interviews.

We were only able to interview four family members who were all from NH2. Our pool of potential family carers to interview was only 15 and eight of these did not respond to the invitation or reminders from the manager. Ethical requirements prevented the research team directly contacting these carers. Of the participants who contacted us, we were able to interview four out of seven carers. A larger sample and representation from NH1 would be required to provide a more in-depth account of the family carers’ views of EOL care conversations. The highly pressured environment of the NH where most interviews took place also restricted the time that participants, particularly staff, had available to complete the interview. Many interviews were therefore short reducing our capacity to explore issues in-depth.

The ICL being researcher and the individual having discussions could have created a bias in reflections towards favouring the *Intervention*. However, our findings did not identify any beneficial aspects of the ICL role that were not supported by the interviews and the purpose of this paper was not to test the *Intervention* but to explore the process of EOL conversations in this context. Another possible limitation is that NH staff and family members may have been inhibited as a result of being observed by the ICL.

Another limitation was the short term nature of this study as the *Intervention* was only implemented for six months. Longer duration of the ICL role may have led to greater NH staff development and confidence in undertaking EOL conversations with family members. However, there is some evidence in the literature that increasing the duration of interventions without adapting their implementation to the context of the particular setting may lead to a decay in adherence and effectiveness, sometimes referred to as ‘programme drift’ and ‘voltage drop’ [53]. Lastly, these findings may only reflect families and staff in the two NHs in Greater London and may not be generalised or reflect practice elsewhere in the UK or internationally, especially in the USA where ACPs are part of routine care [54, 55].

### Clinical implications and further research

UK guidelines indicate that ACP discussions should be carried out with individuals with dementia before they lose capacity to take part in these discussions [11] and that these discussions should be conducted in primary care by a trained individual with appropriate skills [56]. Often it is considered the GP’s role to initiate these conversations, with little acknowledgment that conversations may occur in secondary care. As illustrated in this study, the lack of ownership means that when someone moves into a NH these conversations have usually not taken place. The role of NH staff in these discussions warrants further attention. There may be benefits of a designated role to communicate and discuss EOL with family members and providing time and space to develop trusting relationships. This role could be carried out by NH staff, however, staff in this study lacked confidence and communication skills to implement these discussions and HCPs believed that the role should be undertaken by those with palliative and/or dementia expertise such as Admiral Nurses or, given sufficient resources, an ICL role. Costs of employing an independent ICL have been explored in a naturalistic study (Moore K, Candy B, Davis S, Gola A, Harrington J, Kupeli N, Vickerstaff V, King M, Leavey G, Nazareth I, Omar RZ, Jones L, Sampson EL: Implementing the Compassion Intervention, a model for integrated care for people with advanced dementia towards the end of life in care homes: An exploratory, naturalistic study, in preparation) but would need further investigation in more extensive pilot work. An individual, independent of the NH, appears to be beneficial for both staff and families in that they were perceived not to have vested interests in how care was provided. However, given the growing number of people with dementia residing in nursing homes, the need for NH nurses to undertake this role in the future may be necessary. Interventions that help staff to take on this role, addressing communication and time barriers need to be cultivated.

Further research could explore educating professionals and families together as this may enable the two groups to understand one another’s point of view and allow families to share their stories and concerns [57]. Research could also explore the long term effectiveness of the ICL role or other external individuals such as social workers to support EOL discussions. Having multidisciplinary input involving NH staff and external HCPs could help improve care management and treatment decisions and ensure residents’ and family members’ EOL requests are met [17, 35]. The implementation of an ICL or similar role in NHs could be commissioned within existing palliative care or community mental health teams, or may be commissioned through agencies in the non-profit sector. If this role is commissioned, an ICL requires mechanisms of support, communication and governance for their role.

## Conclusion

While policies advocate early decision making in dementia care, in practice this rarely happens. Despite barriers to discussions, families were able to recognise the importance of having discussions to reassure, support and educate them. This study highlights EOL discussions should be ongoing rather than a one-off task driven conversation. Conversations cannot be rushed and require appropriate time and space to develop sensitively. Having a coordinator similar to the ICL role, who has the time, knowledge and communication skills to have EOL discussions with family and train and support staff, offers a way forward to promoting these discussions in the nursing home setting.

## Abbreviations

ACP, Advance Care Plan; DNAR/DNR, Do Not Attempt Resuscitation; EOL, End–of-life; GP, General Practitioner; HCP, Healthcare professional; ICL, Interdisciplinary Care Leader; MDT, Multidisciplinary Team; NH, Nursing home.
